# Generation of human induced pluripotent stem cells by simple transient transfection of plasmid DNA encoding reprogramming factors

**DOI:** 10.1186/1471-213X-10-81

**Published:** 2010-08-03

**Authors:** Karim Si-Tayeb, Fallon K Noto, Ana Sepac, Filip Sedlic, Zeljko J Bosnjak, John W Lough, Stephen A Duncan

**Affiliations:** 1 Department of Cell Biology, Neurobiology and Anatomy, The Medical College of Wisconsin, 8701 Watertown Plank Road, Milwaukee, WI, 53226, USA; 2 Department of Anesthesiology, The Medical College of Wisconsin, 8701 Watertown Plank Road, Milwaukee, WI, 53226, USA

## Abstract

**Background:**

The use of lentiviruses to reprogram human somatic cells into induced pluripotent stem (iPS) cells could limit their therapeutic usefulness due to the integration of viral DNA sequences into the genome of the recipient cell. Recent work has demonstrated that human iPS cells can be generated using episomal plasmids, excisable transposons, adeno or sendai viruses, mRNA, or recombinant proteins. While these approaches offer an advance, the protocols have some drawbacks. Commonly the procedures require either subcloning to identify human iPS cells that are free of exogenous DNA, a knowledge of virology and safe handling procedures, or a detailed understanding of protein biochemistry.

**Results:**

Here we report a simple approach that facilitates the reprogramming of human somatic cells using standard techniques to transfect expression plasmids that encode OCT4, NANOG, SOX2, and LIN28 without the need for episomal stability or selection. The resulting human iPS cells are free of DNA integration, express pluripotent markers, and form teratomas in immunodeficient animals. These iPS cells were also able to undergo directed differentiation into hepatocyte-like and cardiac myocyte-like cells in culture.

**Conclusions:**

Simple transient transfection of plasmid DNA encoding reprogramming factors is sufficient to generate human iPS cells from primary fibroblasts that are free of exogenous DNA integrations. This approach is highly accessible and could expand the use of iPS cells in the study of human disease and development.

## Background

Human iPS cells are potentially valuable tools for the study of human development and disease. If human iPS cells are generated from specific patients they offer an opportunity to study the molecular mechanism underlying the pathogenesis [1,2]. Because human iPS cells can be derived from a patient's own cells they may ultimately provide the means for effective cell therapy, avoiding concerns associated with immune rejection. However, before iPS cell-based therapeutics can be realized, it is important that a reliable, reproducible, accessible, and safe reprogramming protocol is adopted.

Current human iPS production techniques have several limitations that restrict their clinical usefulness. For example, most reprogramming procedures result in clones of iPS cells in which the extent of reprogramming can be heterogeneous. The derivation of fully reprogrammed iPS cells, therefore, requires fastidious attention to detail with laborious, time-consuming production and screening procedures. Recent studies have suggested that the inclusion of Valproic Acid or Sodium Butyrate may enhance complete reprogramming [3,4].

In addition to the heterogeneity associated with reprogramming, the most commonly used reprogramming protocols use lentiviruses, which integrate into the host cell's genome and are potentially mutagenic. The choice of using lentiviruses to reprogram is partly historical. Initial studies showed that the use of lentiviruses to transduce exogenous factors (*OCT4*, *SOX2*, *NANOG *and *LIN28 *or *OCT3/4*, *SOX2*, *KLF4 *and *C-MYC*) into genomic DNA was sufficient to reprogram human somatic cells [5,6]. Since expression of the viral cDNAs was repressed by methylation this allows transient expression of reprogramming factors until endogenous regulators of pluripotency take over. The transient expression of reprogramming factors may be important because even subtle changes in expression of these genes can induce cell differentiation [7]. Studies using mouse cells have shown that expression of the exogenous reprogramming factors is required for a minimum of 12 days to produce iPS cells [8]. Although the need for extended expression of introduced cDNAs suggested that the use of non-integrating approaches would be challenging, Stadtfeld *et al. *demonstrated that genomic integration was not essential to successfully reprogram mouse fibroblasts, by using adenoviruses to supply cDNAs encoding reprogramming factors [9].

Recently, a number of publications [10,11] have reported the production of human iPS cells that are free of transgenic sequences using a variety of strategies including infection of cells with recombinant adenoviruses and sendai viruses. Although the use of these viruses circumvent genomic integration of DNA, they require a knowledge of virology to produce high titer viruses and adherence to increasingly strict biosafety regulations. Kim *et al *[12] were able to generate human iPS cells by directly delivering reprogramming proteins (OCT4, SOX2, KLF4 and C-MYC). The reprogramming proteins were fused to a peptide highly enriched in basic amino acids, which allowed them to cross the cell membrane barrier. The cells were then seeded on mouse feeder cells, and human iPS-like colonies were picked 56 days after first exposure to the reprogramming proteins. This protocol was advantageous as it did not directly use genetic material to reprogram the somatic cells. However, one limitation was that a cell extract of stably transfected HEK-293T cells had to be added once a week for six weeks.

A number of reports have shown that nucleic acids that do not integrate into the recipient genome can be used successfully to make iPS cells. Yu *et al *[13] demonstrated that human iPS cells could be generated from a single nucleofection of three oriP/EBNA1 (Epstein-Barr nuclear antigen-1)-based episomal vectors coding for seven factors (OCT4, SOX2, NANOG, LIN28, KLF4, C-MYC, and SV40Tag), after drug selection and seeding on mouse feeder cells. However, although the presence of oriP/EBNA1 ensured maintenance of the plasmids even after reprogramming, all human iPS cells generated by this approach therefore had to be subcloned to isolate human iPS cell lines void of vector DNA.

While all of these techniques could be valuable, they all suffer from a lack of simplicity and in some cases require substantial knowledge of virology or protein biochemistry. We reasoned that an ideal reprogramming approach would rely on the use of elementary procedures that were readily accessible to any lab with even rudimentary experience in molecular biology. Since Yu and colleagues [13] had successfully used nucleofection with episomal vectors, we addressed whether simple transient transfection of plasmid DNA could generate human iPS cells. We show that two sequential transfections of non-episomal plasmids that independently encode four reprogramming factors (OCT4, NANOG, SOX2 and LIN28) are sufficient to generate human iPS cells. In addition to exhibiting pluripotent cell features, the resulting integration-free human iPS cells have the ability to generate hepatocyte-like cells and cardiac myocytes following directed differentiation in culture.

## Results

### Generation of integration-free virus-free human induced pluripotent stem cells

**Figure 1:**
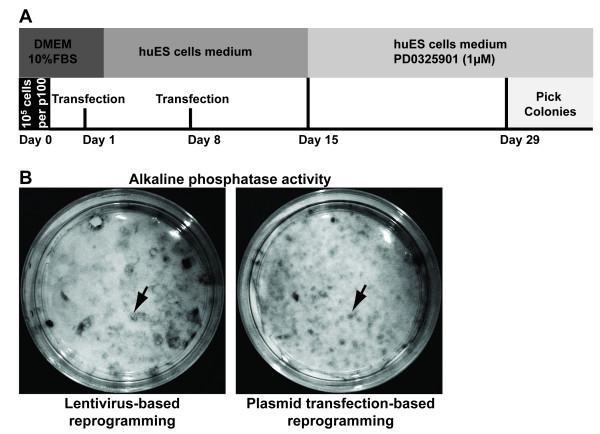
**Overview of approach used to generate iPS cells by transient transfection of plasmid DNA**. A) Schematic and timeline of the protocol used to generate human iPS cells by sequential transient transfection of plasmids expressing OCT4, NANOG, SOX2, and LIN28 cDNAs. B) Photographs showing colonies expressing alkaline phosphatase activity after lentivirus-based reprogramming (left) or plasmid transfection-based reprogramming (right) of human fibroblasts.

Human foreskin fibroblasts previously used for generating lentiviral-derived human iPS cells [13,14] were co-transfected with four plasmid constructs coding for OCT4, NANOG, SOX2 and LIN28 (Figure 1A). Our protocol used plasmid vectors identical to those used to generate the lentiviruses described by Yu *et al *[6]; however, the absence of packaging vectors from the transfection prevented the possibility of generating virions. A second transfection was carried out one week after the first. Two weeks later, transfected cells were incubated with the MEK inhibitor PD0325901 (StemGent). Four weeks after the first transfection, human iPS-like colonies were picked.

At the end of the reprogramming protocol, alkaline phosphatase activity was evaluated (Figure 1B). Compared to the lentivirus-based reprogramming protocol, plasmid transfection led to a similar number of colonies displaying alkaline phosphatase activity. However, even though alkaline phosphatase activity is considered to be a marker of pluripotency in embryonic stem cells [15], it is detected early in the reprogramming process and does not necessarily reflect a fully reprogrammed state [8].

**Figure 2:**
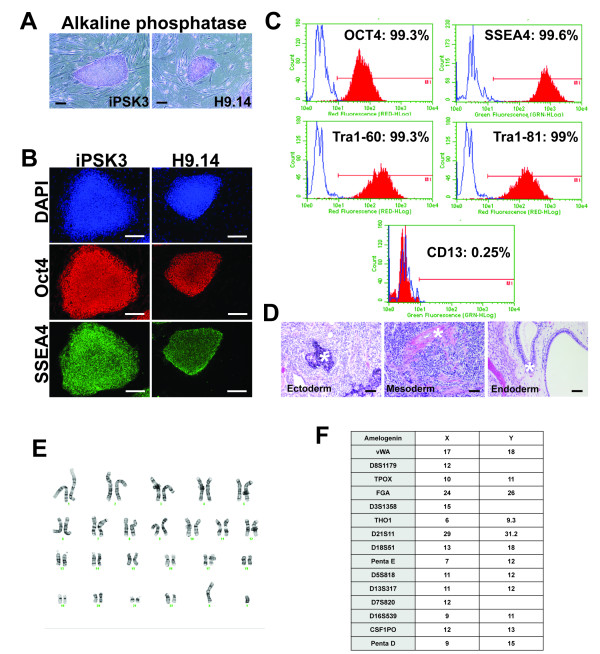
**Analysis of pluripotency of iPS cells generated by plasmid transfection**. A) Micrographs comparing the morphology of the plasmid-derived iPS cells (iPSK3) and human ES cell line H9.14 cultured on mitotically inactivated MEFs and alkaline phosphatase activity identified by histochemistry (Scale bar = 100 μm). B) Immunostaining revealing the presence of OCT4 (red) and SSEA4 (green) in plasmid-derived iPSK3 cells and control H9 human ES cells. Cell nuclei were detected using DAPI stain (Blue) (Scale bar = 100 μm). C) Representative FACS analysis demonstrating that ≥ 99% of iPSK3 cells in culture (passage 8-10) express markers of pluripotency including OCT4, SSEA4, TRA1-60 and TRA1-81. Cells expressing the fibroblast marker CD13 were not detected. D) Micrographs of H & E stained sections through teratomas that formed from iPSK3 cells after injection into immune deficient mice. Cell types derived from all three germ layers - ectoderm, endoderm and mesoderm (indicated with *) - were detected (Scale bar = 100 μm). E) Karyotype of iPSK3 cells revealed a normal distribution of 46 chromosomes with XY sex chromosomes. Chromosomal rearrangements were not detected by G-banding. F) STR analyses using CODIS primers demonstrated that the DNA fingerprint of iPSK3 cells was indistinguishable from that of CRL2097 foreskin fibroblasts used as recipients for reprogramming.

In three independent experiments a total of 36 colonies were collected whose morphology resembled that of huES cells. Cells were collected by manual dissection and seeded onto MEFs. Although we were unable to culture the majority of the colonies as stable lines, a single human iPS cell colony (iPSK3) was successfully expanded for further analyses. As shown in Figure 2A, iPSK3 cells displayed a morphology that was indistinguishable from human embryonic stem cells (H9). The cells also possessed alkaline phosphatase activity (Figure 2A) and expressed pluripotent markers including OCT4 and SSEA4 (Figure 2B). In addition, FACS analyses revealed that ≥ 99% of cells expressed OCT4, SSEA4, TRA1-60 and TRA1-81, while in contrast the fibroblast marker CD13 was not detected (Figure 2C). After subcutaneous injection into immunodeficient Rag2^-/-^; Il2rgamma^-/- ^mice, iPSK3 cells generated teratomas containing tissues derived from all three germ layers (Figure 2D). Finally, G-banding of genomic DNA revealed a normal karyotype (Figure 2E) and Short Tandem Repeat analysis found that the DNA fingerprint of iPSK3 cells matched that of the human foreskin fibroblast originally used for reprogramming (Figure 2F).

**Figure 3:**
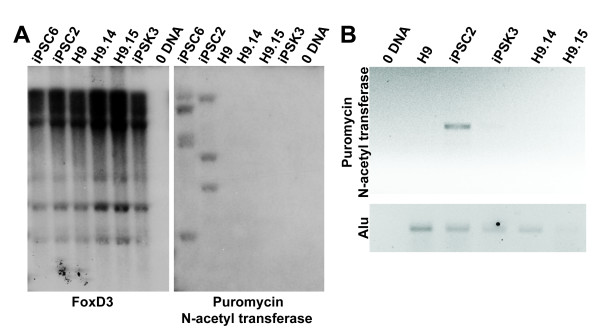
**Human iPSK3 cells are devoid of plasmid DNA**. A) Southern blot analysis of genomic DNA from control iPS cells, iPSC6 and iPSC2 [14], generated by lentivirus transduction, human ES cells (H9, H9.14, and H9.15), or iPSK3 cells. DNA was digested with *BamH1 *and blots were hybridized with probes to detect *FOXD3 *as a loading control (left) or the *puromycin N-acetyl transferase *gene (right), which is present in both plasmid and lentiviral vectors. B) PCR analysis on total DNA extracted from either human ES cells (H9, H9.14 and H9.15), which lack exogenous DNA sequences, iPSC2 cells which contain integrated viral sequences, and iPSK3 cells using primers that specifically recognize the *puromycin N-acetyl-transferase *gene. PCR amplification without template DNA was performed to exclude DNA contamination. Primers that specifically recognize a human Alu sequence were used for loading control.

Since the plasmids used to reprogram lacked sequences required for replication in eukaryotic cells we expected that exogenous DNA would by lost from iPSK3 cells during cell division. We, therefore screened for the presence of plasmid DNA by Southern blot and PCR analysis of iPSK3 genomic DNA. Southern blot analysis was performed after digestion by BamHI and developed using a probe to detect the *puromycin N-acetyl transferase *gene that is present on each of the plasmids used for reprogramming but is absent from human genome (Figure 3A right). Blots were stripped and hybridized to a probe that detected *FOXD3*, to ensure the presence of DNA in each sample (Figure 3A left). While integrated exogenous DNA was detected in lentiviral-derived human iPS cells (iPSC2 and iPSC6), plasmid DNA was not detected in control huES cell lines H9, H9.14 and H9.15 (two subclones of H9) or in iPSK3 cells. Since genomic DNA was obtained by precipitation from whole cell lysates, without separation between cytosolic and nuclear compartments, the absence of exogenous DNA by Southern blot suggests that iPSK3 cells were also devoid of episomal plasmid DNA (Figure 3A).

The absence of plasmid DNA was confirmed by performing genomic PCR using primers that specifically amplify puromycin N-acetyl-transferase sequence (Figure 3B). An amplification product was successfully detected when DNA from control lentiviral-derived iPSC2 cells was used as a template. In contrast, amplicons were not identified in DNA from control H9 cells or from iPSK3 cells. From these data we conclude that the reprogrammed iPSK3 cells are devoid of plasmid sequences, at least within the resolution offered by the assays.

### Differentiation of iPSK3 cells into hepatocyte-like cells

**Figure 4:**
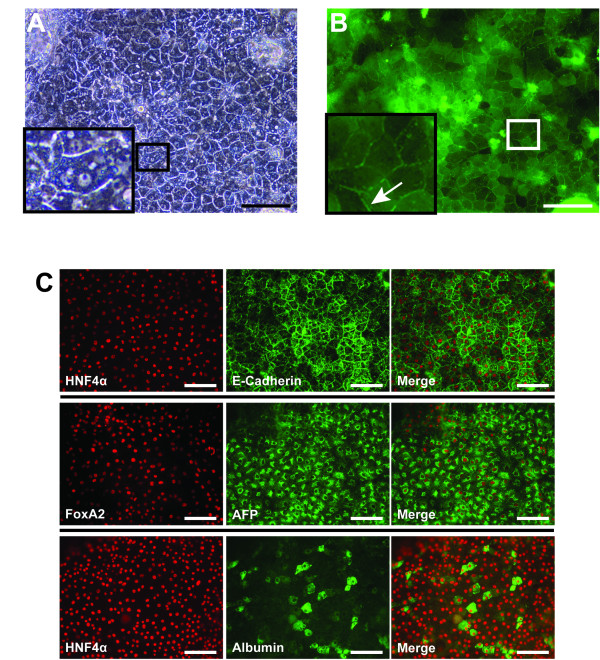
**Differentiation of iPSK3 into hepatocyte-like cells**. A) Micrograph showing the epithelial-like organization of iPSK3 upon hepatic differentiation. Higher resolution (inset) showing a binucleated cell. Scale bar = 100 μm. B) The hepatocyte-like cells derived from iPSK3 metabolized and transported 5-(and 6)-carboxy-2'-7'-dichlorofluorescein diacetate between cells (high-resolution inset, white arrow), indicating the presence of functional bile transporters. C) Immunocytochemistry revealed the presence of proteins commonly associated with hepatocytes including, FoxA2, Hnf4α, E-cadherin, α-fetoprotein and Albumin. Scale bar = 100 μm.

We previously reported that human iPS cells can be used to efficiently generate cells that possess features and functional characteristics that are similar to those of human hepatocytes [14]. We, therefore, tested whether iPSK3 cells retained competency to adopt a hepatic fate by directed differentiation in culture. Following completion of the four-step differentiation protocol [14], cells derived from iPSK3 cells displayed features that are associated with hepatocytes. Such features included an epithelial organization, large cytoplasmic-to-nuclear ration, prominent nucleoli, the presence of lipid vesicles, and the presence of binucleated cells in the culture (Figure 4A, see insert).

Differentiated cells were then incubated with 5-(and 6)-carboxy-2'-7'-dichlorofluorescein diacetate (DCF-DA). DCF-DA is membrane permeable and becomes trapped inside the cells after cleavage by intracellular esterase into DCF and DA. DCF, however, can be secreted between hepatocyte-like cells via bile canaliculi through specific transporters [16]. Hepatocyte-like cells derived from iPSK3 efficiently secreted the fluorescent compound, demonstrating the presence of functional bile transporters (Figure 4B; see inset, white arrow). Finally, the differentiated cells also expressed several hepatic markers including FoxA2, E-cadherin, HNF4α, α-fetoprotein, and Albumin (Figure 4C). Overall, our data suggest that the iPSK3 were competent to form hepatocyte-like cells.

### Directed differentiation of human iPSK3 cells into cardiac myocytes

**Figure 5:**
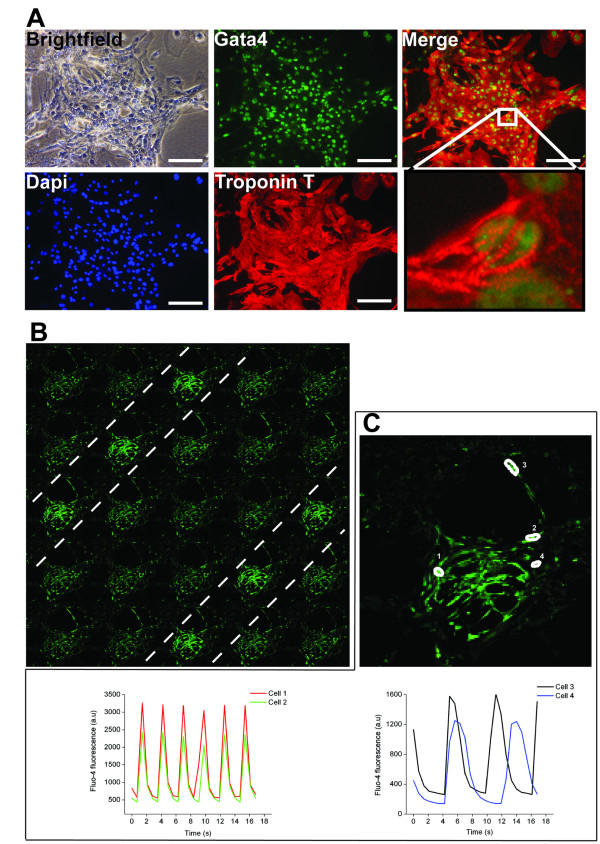
**Differentiation of iPSK3 cells into cardiac cells**. A) Immunocytochemistry revealed that clusters of contracting cells expressed cardiac markers including GATA4 in the cells nuclei, detected by DAPI staining, and cardiac-specific Troponin T. High-resolution images revealed a striated pattern of Troponin T staining as expected for a protein associated with myofibrils (Scale bar = 100 μm). B) Intracellular calcium concentration measurements using the fluo-4 AM fluorescent indicator identified rhythmic oscillation correlating with cardiac cell contraction (0.7s between each picture; see movie S2 in supplemental data). C) Contracting cells within the cluster exhibit both an atrial-like calcium transient (cell 1 and 2, lower left panel) and a ventricular-like calcium transient (cell 3 and 4, lower right panel).

To ensure that the cells' ability to differentiate in culture was not restricted to cells derived only from endodermal lineages, we examined the potential of the iPSK3 cells to differentiate toward a cardiac myocyte cell fate using a directed cardiac myocyte differentiation protocol based on previously published procedures [17,18]. Clusters of rhythmically contracting cells were observed 2 weeks following the start of the differentiation protocol and continued to beat 40 days later (Additional file 1). The beating areas expressed the transcription factor GATA4 (Figure 5A) and the cardiac-specific marker Troponin-T in a striated manner, consistent with these cells being cardiac myocytes (Figure 5A and inset).

Cytosolic calcium transients were monitored using the fluo-4 AM fluorescent indicator, which is calcium sensitive. Figure 5B shows a montage of rhythmic oscillation of intracellular calcium waves based on video acquisition (Additional file 2). Analysis of intracellular calcium released from individual cells among the beating network (Figure 5C, upper) showed a heterogeneous population consisting of atrial-like cardiac myocytes (Figure 5C, lower left) that displayed a higher frequency of intracellular calcium oscillation and shorter periodicity, along with ventricular-like cardiac myocytes (Figure 5C, lower right), with a lower frequency of intracellular oscillation and longer periods. These data confirm that iPSK3 cells can be induced to differentiate into distinct populations of functional cardiac myocytes in culture.

## Discussion

In this report, we describe the generation of human iPS cells devoid of exogenous DNA by the simple transient transfection of plasmids that express reprogramming factors. These newly reprogrammed somatic cells display pluripotent markers and were found to form teratomas in immunodeficient mice that were composed of all three germ layers. In addition, our data demonstrate that the iPSK3 cells produced by this process could form hepatocyte-like cells and contracting cardiac myocytes upon directed differentiation in culture.

Despite the observation that similar numbers of colonies expressing alkaline phosphatase activity were identified between lentiviral transduction and plasmid transfection, the use of plasmids is less efficient for full reprogramming of somatic cells. Based on the successful isolation of a single fully reprogrammed colony, the efficiency of human iPS cell reprogramming by plasmid transfection (1 iPS cell from 300,000 fibroblasts) was similar to that reported for the use of adenovirus (0.0002%) [10], which is approximately ten-fold lower (0.01%) when reprogramming is performed with lentiviruses [6]. This lower efficiency presumably reflects a reduction in sustainable expression of the reprogramming factors because the presence of plasmid DNA within the cells is transient. A substantial effort by multiple labs has resulted in the description of small molecules that can enhance the efficiency of reprogramming (See review by Li and Ding [19]). For example increases up to 200-fold in reprogramming efficiency have been reported by including antagonists of TGFbeta signaling in addition to MEK-ERK inhibitors and passaging the cells in the presence of Thiazovivin [20]. In addition, inclusion of Valproic Acid or Sodium Butyrate may enhance complete reprogramming [3,4]. It therefore seems likely that such drug combinations could be used to increase the efficiency of reprogramming by transient transfection of expression plasmids.

The main advantage of the current approach is that it allows direct reprogramming of human somatic cells into human iPS cells devoid of exogenous DNA without the need for extensive experimental procedures [12,13]. While several other procedures have been described for producing iPS cells free of integrating DNA, most require some level of specialized knowledge of virology or biochemistry. Although the frequency of recovering completely reprogrammed iPS cells is lower than using lentiviral transduction, the simplicity and accessibility of using transient transfection should also compensate for the reduced efficiency.

## Conclusions

In conclusion, we have shown that simple transient transfection of plasmid DNA encoding reprogramming factors is sufficient to generate human iPS cells from primary fibroblasts that are free of exogenous DNA integrations. This approach is simple, highly accessible and could expand the use of iPS cells in the study of human disease and development, as well as increase the availability of safer iPS cells for potentially therapeutic uses.

## Methods

### Cell culture

Human iPS cells were cultured on mitotically inactivated mouse embryonic fibroblasts (MEFs) in a stem cell medium composed of Dulbecco's modified Eagle's medium/Ham's F-12 (DMEM/F12) supplemented with 20% knockout serum (Invitrogen, Carlsbad, CA), non-essential amino acids solution (Invitrogen, Carlsbad, CA), glutamine (Invitrogen, Carlsbad, CA), penicillin/streptomycin (Invitrogen, Carlsbad, CA) and bFGF (4 ng/ml; Invitrogen, Carlsbad, CA) and maintained under low oxygen conditions (4% O_2_; 5% CO_2_) for 7 days. Cells were manually passaged once a week on MEFs, by using Accutase (Millipore, Billerica, MA) on Matrigel, or on a recombinant protein substrate (StemAdhere™, Primorigen Inc, Madison, WI) as described previously [21]. For differentiation experiments, MEF-conditioned medium was used to maintain pluripotency while transferring the cells onto feeder cell-free Matrigel-coated plates. All work carried out using human iPS cells was approved by the MCW Human Stem Cell Research Oversight Committee (hSCRO approval# 09-005) committee and all work performed using animals was approved by the MCW IACUC.

### Reprogramming protocol

On day 0, 1 × 10^5 ^human foreskin fibroblasts (ATCC cell line CRL2097) were plated on an uncoated 100 mm dish. To introduce DNA, pSin vectors coding for OCT4, NANOG, SOX2, and LIN28 [6] were mixed with Fugene 6 (Roche Applied Science, Indianapolis, IN) in a 2:1 ratio of Fugene:DNA and transfections were performed on day 1 and 8 of reprogramming. Stem cell medium was used for all cell culture starting on day 2 of reprogramming, and the MEK1 inhibitor PD0325901 (StemGent, Cambridge, MA) was added to the medium on day 15 at a final concentration of 1 μM. The medium was changed daily. After four to five weeks, emerging colonies were manually collected and plated on mitotically-inactivated MEFs for subsequent characterization. Lentivirus-based reprogramming utilized the same culture conditions as described above; however, the cells were infected with concentrated replication-incompetent pseudotyped lentiviruses that express OCT4, NANOG, SOX2 and LIN28.

### Alkaline Phosphatase activity

Pluripotent cells in culture on MEFs were fixed with 4% paraformaldehyde for 2 min at room temperature. The cells were then rinsed with TBST (20 mM Tris-HCl, pH 7.4, 0.15 M NaCl, 0.05% Tween-20) and incubated for 15 min at room temperature with a mixture of Fast Red Violet, Naphtol AS-BI phosphate solution and water (2:1:1) from the Alkaline Phosphatase Detection Kit (Millipore, Billerica, MA) according to the manufacturer's protocol. Following staining, the cells were rinsed with TBST and covered with phosphate-buffered saline (PBS).

### Karyotyping and DNA fingerprinting

Karyotyping and DNA fingerprinting of the human induced pluripotent cell lines was performed by Cell Line Genetics, Madison, WI (http://www.clgenetics.com/).

### Teratoma assay

Human iPS cell line, iPSK3, was cultured on MEFs with one dense 60 mm plate used per assay. Cells were manually removed using the StemPro EZPassage tool (Invitrogen), collected by centrifugation and resuspended in 250 μl of Matrigel (2 mg/ml in DMEM-F12; BD Bioscience). Approximatively 2 × 10^6 ^cells were then injected into the posterior dorsal side of immunodeficient Rag2^-/-^; Il2rg^-/- ^mice using a 27G needle. Resulting teratomas were dissected 8 to 9 weeks after injection, fixed overnight with Zinc-formalin and embedded in paraffin before 4 μm sections were stained with hematoxilin and eosin. All animal procedures were approved by the MCW IACUC.

### Immunostaining

Cultured cells were fixed with 4% paraformaldehyde for 30 min at room temperature, permeabilized with 0.5% Triton X-100 in PBS for 15 min and blocked with 3% BSA in PBS for 15 min. Cells were incubated overnight at 4°C with primary antibodies diluted in 1% BSA in PBS (Oct3/4 1:500; SSEA4 1:100; GATA4 1:500; Cardiac Troponin T 1:100; FoxA2 1:250; Hnf4a 1:500; E-cadherin 1:1000; alpha-fetoprotein 1:500; Albumin 1:500). After incubation with respective secondary antibodies (Alexa Fluor 488 or 568; Invitrogen, Carlsbad, CA) for 1 h at room temperature, cell nuclei were identified using DAPI and samples were mounted using ProLong Gold Antifade Mounting Reagent (Invitrogen, Carlsbad, CA).

### Flow Cytometry analysis

Cultured cells were dissociated using a PBS-based Cell Dissociation Buffer (Invitrogen), washed with 0.25% BSA in PBS and stained 1 hour with antibodies directed against CD13-Alexa 488 nm, OCT3/4-PerCP, SSEA4-Alexa 488 nm, TRA1-60-PE or TRA1-81-PE (CD13 1 μg; Oct3/4 1 μg; SSEA4 0.5 μg; TRA1-60 1 μg; TRA1-81 1 μg) at 4°C. Flow cytometry analysis was performed using the Guava EasyCyte Minisystem (Millipore, Billerica, MA).

### Southern blot and PCR analysis

The cells were incubated overnight with a cell lysis buffer (4 M urea, 10 mM CDTA, 0.5% sarkosyl, 0.1 M Tris-HCL, pH 8.0, 0.2 M NaCl) and 1 mg/ml proteinase K at 55°C. The next day, isopropanol was added and the DNA was spooled out and dissolved in 10 mM Tris-HCL, pH 8.0, 0.1 mM EDTA. Genomic DNA was digested with BamHI and resolved on a 0.7% agarose gel at 23 V overnight. After denaturing, DNA from the gel was transferred to a Zetaprobe GT membrane (Bio-Rad, Hercules, CA). Fragments were amplified by PCR to generate a radioactively-labeled probe that recognized puromycin N-acetyl-transferase (which confers resistance against puromycin in eukaryotic cells) or *FOXD3 *as a control for all DNA samples. PCR analysis was performed on 2 ng of DNA using specific primers for *puromycin N-acetyl-transferase *(Puro) and human *Alu *repeat as a loading control. Primers used to generate probes and amplicons: Puro, GTCACCGAGCTGCAAGAACT and GTCCTTCGGGCACTCGAC; *FOXD3*, GTGAAGCCGCCTTACTCGTAC and CCGAAGCTCTGCATCATGAG; *Alu*, AATATGGCCCAACTGCAGAA and CATCGCATTTTCACATCCAA.

### Hepatocyte-like cell differentiation

Pluripotent cells were differentiated using the method described previously [14]. To assess cell polarity hepatocyte-like cells were incubated for 10 min with 5-(and 6)-carboxy-2'-7'-dichlorofluorescein diacetate (DCF-DA; Biotium) at a final concentration of 2 μg/ml. Cells were then washed three times with hepatocyte culture medium and immediately observed under a fluorescent microscope.

### Cardiac myocyte differentiation

Pluripotent cells were manually dissected from MEFs and plated on 6-well plates pre-coated with Matrigel and kept under 4% O_2_/5% CO_2 _at 37°C for 1 week. To induce cardiac differentiation, cells were incubated for 5 days in RPMI/B27 medium (Invitrogen, Carlsbad, CA), supplemented with 50 ng/ml human recombinant Activin A (R & D systems, Minneapolis, MN) and 10 ng/ml BMP4 (Peprotech, Inc, Rocky Hill, NJ) in a 4% O_2_/5% CO_2 _atmosphere. After 5 days, treated cells were then placed in a 5% CO_2 _and ambient oxygen atmosphere, and the RPMI/B27 medium was changed every 2 days. Clusters of beating cells were observed 15-20 days after starting the differentiation process.

### Intracellular calcium measurements

Intracellular calcium transients in human iPS cell-derived cardiac myocytes were measured using fluo-4 AM fluorescent indicator (Invitrogen, Carlsbad, CA) in RPMI/B27 medium. Fluo-4 AM (2 μM) was loaded for 20 min at 37°C, followed by a 5 min dye washout. Rhythmically contracting areas were visualized using a laser-scanning confocal microscope (Eclipse TE2000-U; Nikon, Tokyo, Japan) with a Plan Apo 10× air NA 0.45 objective lens (Nikon Corp, Tokyo). Images were acquired every 700 ms using EZ-C1 software (EZ-C1 2.10, Nikon Corp, Tokyo), and data were analyzed with MetaMorph 6.1 software (Universal Imaging, West Chester, PA).

## Authors' contributions

KST conducted the experimental design and execution, draft of manuscript, and analyses of data. FKN performed the Southern blot experiment and analyses. AS oversaw the cardiac myocyte differentiation protocol and calcium imaging experiment. FS performed the calcium imaging of cardiac myocytes and data analysis and ZJB contributed to the experimental design and data interpretation of the calcium imaging experiment. JWL designed and oversaw the cardiac myocyte differentiation protocol. SAD oversaw all aspects of experimental design, data interpretation, and the writing of the final draft of the manuscript. All authors read and approved the final manuscript.

## Supplementary Material

Additional file 1 **Beating cluster of cardiac myocytes derived from iPSK3 cells**. Click here for file

Additional file 2 **Calcium transient visualization of a beating cluster of iPSK3 cell-derived cardiac myocytes in the presence of a fluo-4 AM fluorescent indicator**. Click here for file
